# Differential Functions of Individual Transcription Factor Binding Sites in the Tandem Repeats Found in Clinically Relevant *cyp51A* Promoters in Aspergillus fumigatus

**DOI:** 10.1128/mbio.00702-22

**Published:** 2022-04-25

**Authors:** Sanjoy Paul, Paul E. Verweij, Willem J. G. Melchers, W. Scott Moye-Rowley

**Affiliations:** a Department of Molecular Physiology and Biophysics, Carver College of Medicine, University of Iowa, Iowa City, Iowa, USA; b Department of Medical Microbiology, Radboud University Medical Centre, Nijmegen, The Netherlands; c Centre of Expertise in Mycology Radboudumc/CWZ, Radboud University Medical Centre, Nijmegen, The Netherlands; Universidade de Sao Paulo

**Keywords:** *Aspergillus fumigatus*, azole resistance, drug resistance mechanisms, mutational studies, promoters, transcription factors

## Abstract

Aspergillus fumigatus is the major filamentous fungal pathogen in humans. The gold standard treatment of A. fumigatus is based on azole drug use, but the appearance of azole-resistant isolates is increasing at an alarming rate. The *cyp51A* gene encodes the enzymatic target of azole drugs, and azole-resistant alleles of *cyp51A* often have an unusual genetic structure containing a duplication of a 34- or 46-bp region in the promoter causing enhanced gene transcription. These tandem repeats are called TR34 and TR46 and produce duplicated binding sites for the SrbA and AtrR transcription factors. Using site-directed mutagenesis, we demonstrate that both the SrbA (sterol response element [SRE]) and AtrR binding sites (AtrR response element [ATRE]) are required for normal *cyp51A* gene expression. Loss of either the SRE or ATRE from the distal 34-bp repeat of the TR34 promoter (further 5′ from the transcription start site) caused loss of expression of *cyp51A* and decreased voriconazole resistance. Surprisingly, loss of these same binding sites from the proximal 34- or 46-bp repeat led to increased *cyp51A* expression and voriconazole resistance. These data indicate that these duplicated regions in the *cyp51A* promoter function differently. Our findings suggest that the proximal 34- or 46-bp repeat in *cyp51A* recruits a corepressor that requires multiple factors to act while the distal repeat is free of this repression and provides the elevated *cyp51A* expression caused by these promoter duplications.

## OBSERVATION

Aspergillus fumigatus is the most common cause of mold infections in humans (1). Azole drugs are currently the first-line therapy for aspergillosis. However, azole-resistant A. fumigatus clinical isolates are being found with increasing frequency and are associated with a significantly worse clinical outcome (2). Although multiple mechanisms contribute to azole resistance in A. fumigatus, the most commonly reported genetic changes associated with this phenotype are alterations in the gene encoding Cyp51A, the target enzyme of azole drugs (3). The most prevalent azole resistance allele is a compound mutation in *cyp51A* consisting of a 34-bp duplication in the promoter element (TR34) and a single amino acid replacement in the coding sequence (L98H) (4). Both of these mutations are required for the observed high-level azole resistance conferred by this compound allele (5).

Although it is well established that the TR34 *cyp51A* promoter drives increased expression of *cyp51A* mRNA compared to the wild-type version (5), we lack a detailed understanding of how this increased expression is achieved. Previous studies from our lab and others have demonstrated that several different transcription factors control transcription of *cyp51A* via the 34-bp region. First, the sterol-responsive SrbA regulator binds to an element in this 34-bp region called the sterol response element (SRE) and stimulates expression when sterols are limiting (6, 7). Second, the AtrR transcription factor binds to a second site within the 34-bp region, referred to as the AtrR response element (ATRE), to activate transcription (8, 9). Finally, two different negative transcriptional regulators repress *cyp51A* expression. The CCAAT-binding complex (CBC) and the iron-responsive transcription factor HapX (10) both reduce *cyp51A* expression: CBC binds within the 34-bp region, while HapX binds just 3′ to this segment (11, 12). The locations of these sites and their positions relative to the 34-bp region are shown in Fig. S1A in the supplemental material. Note that both the TR34 and TR46 promoters contain two SREs and ATREs owing to the 34-bp duplication. The CBC binding site is also duplicated, but the HapX response element (HXRE) is not. To distinguish between these two copies of each site, we refer to them as either the proximal SRE/ATRE (proximal; closest to transcription start [pSRE/pATRE]) or distal SRE/ATRE (distal; furthest from transcription start [dSRE/dATRE]).

10.1128/mbio.00702-22.2 FIG S1 Detailed map of *cyp51A* promoter mutations and analysis of Cyp51A protein levels in response to these alterations. (A) The DNA sequence of the *cyp51A* promoter region of interest in this study is shown. The wild-type promoter is shown at the top, and the TR34 equivalent is shown at the bottom. Locations of the core binding elements for each transcription are indicated below the DNA sequences. Mutant bases are shown in red lettering for each site. The extent of the 34-bp repeat is shown by the gray highlighting. (B) Whole-cell protein extracts were prepared and analyzed by Western blotting using the anti-Cyp51A antiserum (S. Paul, D. Diekema, and W. S. Moye-Rowley, Eukaryot Cell 12:1619–1628, 2013, https://doi.org/10.1128/EC.00171-13). Strains lacking the pATRE in the wild-type *cyp51A* promoter context were unable to be grown in the presence of voriconazole and are absent from that analysis. Lanes are numbered at the top of each panel and the numbers near each Cyp51A polypeptide correspond to the quantitation for this experiment. A representative experiment of at least two is shown, and expression levels were all normalized back to the wild-type Cyp51A with no drug treatment. Download FIG S1, PDF file, 0.08 MB. Copyright © 2022 Paul et al. 2022 Paul et al. https://creativecommons.org/licenses/by/4.0/ This content is distributed under the terms of the Creative Commons Attribution 4.0 International license.

**FIG 1:**
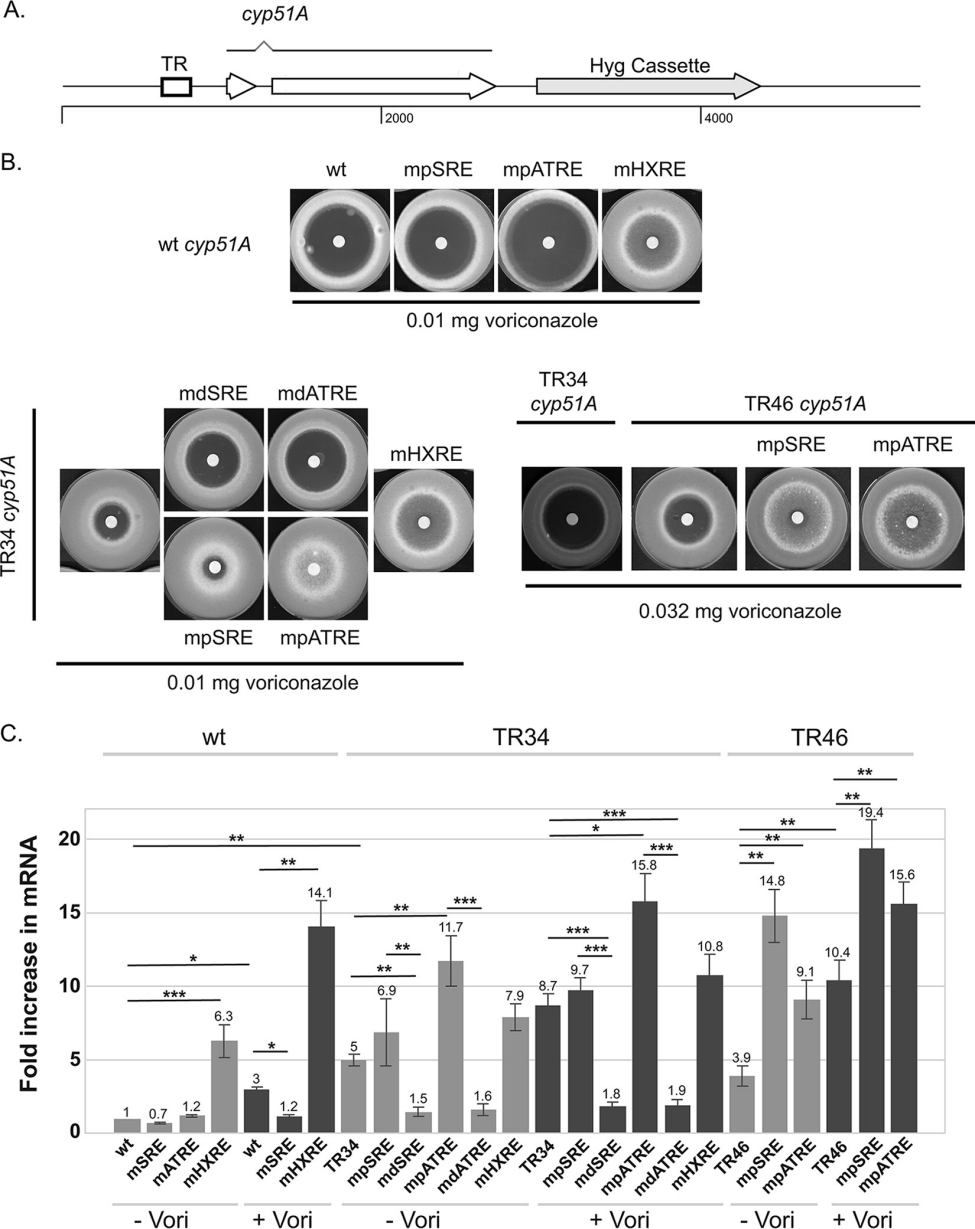
Analysis of *cyp51A* promoter function. (A) Schematic diagram of final structure of wild-type and mutant *cyp51A* promoter mutants. All mutants analyzed were reintroduced back at the native *cyp51A* chromosomal location using CRISPR-based recombination with the insertion of the downstream hygromycin cassette as described earlier (17). The relative location of the TR repeat regions is indicated as a box, with the two exons of *cyp51A* also noted. The hygromycin selection marker (Hyg cassette) is located downstream of the native 3′ end of the *cyp51A* mRNA. (B) Disk diffusion assay of mutant forms of the *cyp51A* promoter. For the wild type (wt) and TR34 derivatives, a filter disk containing 0.01 mg of voriconazole was placed in the center of 10^6^ spores of each indicated strain and allowed to grow at 37°C for 72 h. For TR46 derivatives, the voriconazole amount was increased to 0.032 mg. (C) Strains containing the listed versions of either the wild-type (left-hand side) or TR34 (right-hand side) *cyp51A* gene were grown to mid-log phase with (+) or without (-) voriconazole treatment. Transcriptional behavior of each mutant promoter was assessed by qRT-PCR relative to the *tef1* gene. Data are presented for the average of two independent experiments. Numbers above each bar represent the average fold increase for each strain in the presence or absence of voriconazole. Student’s *t* test was used to compare mRNA changes for the pairs indicated by the lines. Paired conditions were used for the same isolate assayed with and without the drug, while unpaired conditions were used to compare different isolates. Significance is expressed as follows: *, *P* < 0.05; **, *P* < 0.01; and ***, *P* < 0.001.

To evaluate how the ATRE, SRE, and HXRE contribute to expression of both wild-type and TR34 versions of *cyp51A*, site-directed mutations were constructed in these elements (Fig. S1A) and returned to the natural *cyp51A* genomic location (Fig. 1A). These strains were tested for their ability to grow in the presence of voriconazole (Fig. 1B), and the level of *cyp51A* expression was evaluated by reverse transcription-quantitative PCR (RT-qPCR) (Fig. 1C) or using an anti-Cyp51A antibody (Fig. S1B).

Loss of either the pSRE (mpSRE) or the pATRE (mpATRE) from wild-type *cyp51A* caused a slight (mpSRE) or a large (mpATRE) increase in voriconazole susceptibility (Fig. 1B). Removal of the HXRE led to a large decrease in voriconazole susceptibility. These resistance data were fully consistent with the observed expression changes seen by either RT-qPCR measurements (Fig. 1C) or Western blotting (Fig. S1B). Loss of the ATRE from the wild-type *cyp51A* promoter caused such profound hypersensitivity to voriconazole that we were unable to recover sufficient fungus to assay expression. Together, these data are consistent with both the SRE and ATRE acting as positive regulatory elements and the HXRE acting as a negative element to control *cyp51A* expression and function.

Insertion of the TR34 promoter into the *cyp51A* locus led to a decrease in voriconazole susceptibility as seen before (4, 13). Strikingly, loss of either the pSRE or the pATRE from TR34 *cyp51A* led to a large decrease in voriconazole susceptibility (Fig. 1B). This decrease in voriconazole susceptibility was accompanied by a large increase in the level of Cyp51A expression (Fig. 1C; Fig. S1B). The behavior of each of these proximal element mutations was similar to that caused by loss of the HXRE from TR34 *cyp51A*. Although these proximal binding sites clearly work as primarily as positive elements in the wild-type promoter context, they appear to be involved in repression in the TR34 promoter, as their loss leads to a large increase in *cyp51A* expression. Conversely, loss of either of the distal elements (dSRE or dATRE) caused an increase in voriconazole susceptibility, along with a decrease in expression and loss of voriconazole inducibility of *cyp51A* mRNA (Fig. 1C) consistent with these binding sites acting as positive sites determining TR34 promoter function.

We also produced proximal ATRE and SRE mutant forms of the TR46 *cyp51A* gene to determine if the unexpected behavior of these elements would extend to this different promoter context. TR46 corresponds to duplication of 46 bases with an identical 5′ endpoint to TR34 and an additional 12 bp at the 3′ end (14). As seen for their counterparts in the TR34 promoter, loss of either the proximal SRE or ATRE caused a decrease in voriconazole susceptibility and an increase in expression compared to the starting TR46 promoter-containing strain.

These data indicate that the increased Cyp51A expression and reduced voriconazole susceptibility caused by the TR34 or TR46 promoter cannot be explained simply by the increased dosage of the duplicated regions present. The proximal and distal regions have distinct behaviors in the TR34 promoter context and likely in the TR46 promoter as well. The distal 34-bp region behaves strictly as a positive regulator of *cyp51A* transcription, while the proximal element exhibits a negative effect when present in the TR34 promoter. This is quite surprising since loss of the pATRE from the wild-type *cyp51A* promoter yields a strain that cannot grow in the presence of voriconazole. The same behaviors are seen for the pSRE, although this strain grew, albeit slowly, in the presence of voriconazole.

**FIG 2:**
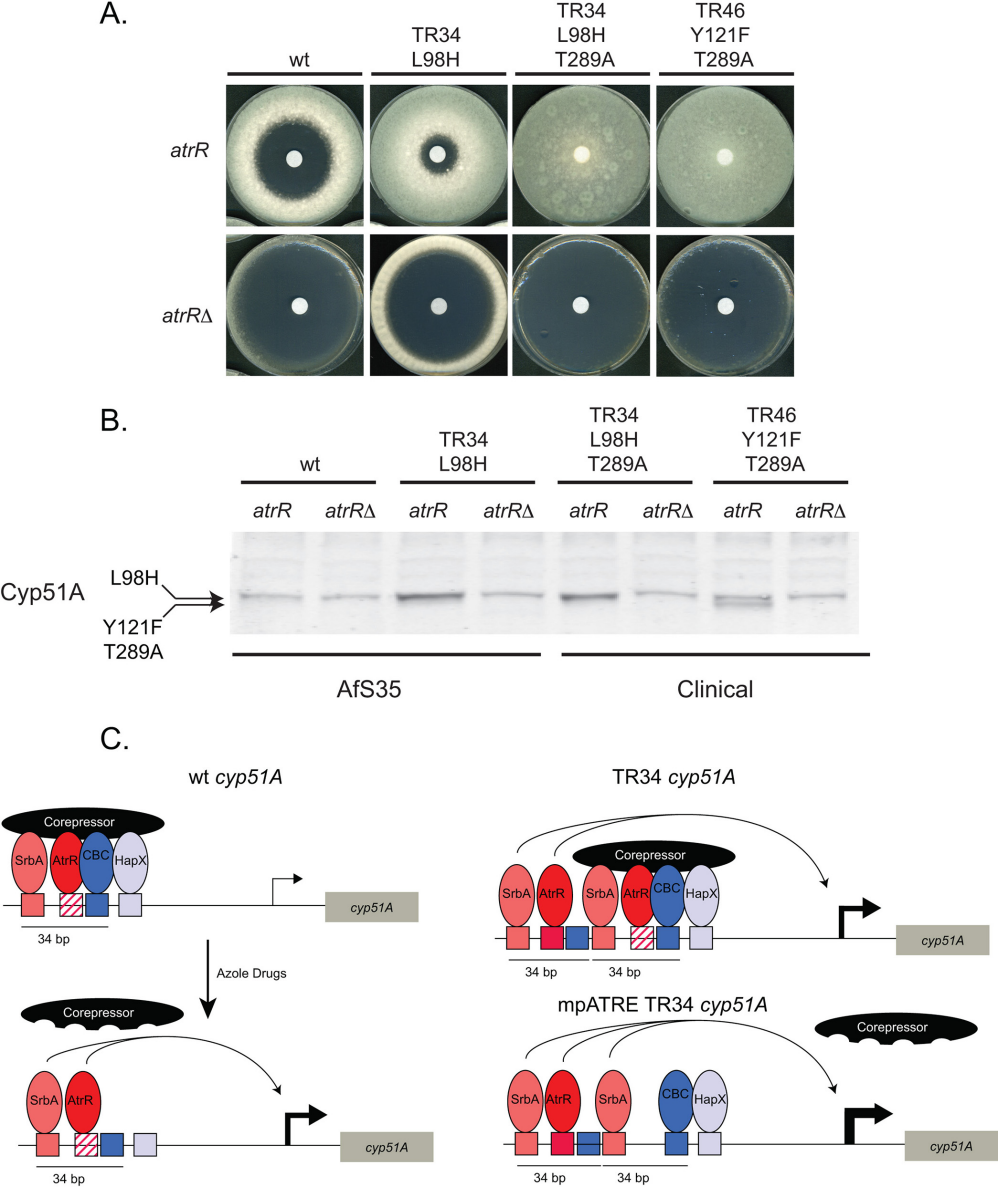
AtrR is essential for voriconazole resistance in laboratory and clinical strains. (A) Isogenic *atrR* and *atrR*Δ derivatives of the indicated strains were tested for voriconazole resistance by disk diffusion assay. (B) Western blot analysis of the strains listed above was performed using anti-Cyp51A antiserum. Note that the L98H-containing enzymes electrophoresed very close to a nonspecific background signal as we showed earlier (13). The Y121F T289A Cyp51A enzyme has a higher mobility and is clearly resolved below this background polypeptide. (C) Diagram for potential roles of *trans*- and *cis*-acting factors at wild-type and TR34 *cyp51A* promoters. A hypothetical corepressor is pictured that makes multivalent contacts with the key regulators of *cyp51A* transcription. The proximal ATRE is indicated by a red hatched box. Other binding sites are color-coded with their respective regulators. Azole drugs trigger corepressor dissociation and gene activation. In the case of the TR34 promoter (right-hand diagrams), the distal SRE and ATRE in the upstream 34-bp repeat can bypass corepressor function and activate transcription. The 34-bp (and 46-bp) tandem repeats do not include the HXRE but maintain a CBC binding site. Interaction of CBC with the adjacent HXRE is required for strong binding of these factors (18). Exposure of the TR34 *cyp51A* gene to azole drugs or loss of the pSRE or pATRE (shown here) triggers strong induction of expression. Induction of expression in the mpATRE TR34 promoter is maximal even in the absence of azole induction. Only TR34 is shown, but we believe that the same mechanisms operate for the TR46 promoter.

Given the important role of AtrR in control of *cyp51A* promoter function, we compared the requirement for this factor in voriconazole resistance and Cyp51A expression in wild-type and isogenic TR34 *cyp51A* laboratory strains. We also examined the effect of loss of AtrR in two different clinical strains containing either a TR34 promoter-driven *cyp51A* gene or a TR46 *cyp51A* locus. Each of the clinical isolates tested is associated with a different mutant form of Cyp51A. The *atrR* gene was disrupted in all these 4 strains using CRISPR/cas9 and isogenic *atrR* and *atrR*Δ derivatives tested for voriconazole susceptibility (Fig. 2A) and expression of Cyp51A by Western blotting (Fig. 2B). Tables 1 and 2 list the strains and plasmids, respectively, used in this study.

**TABLE 1:** A. fumigatus strains used in this study

Strain	Parent	Genotype	Source or reference
V232-12		TR34 L98H T289A *cyp51A*	W. Melchers
SPF169	V232-12	*atrR*Δ::*ptrA*	This study
V181-51		*TR46 Y121F T289A cyp51A*	W. Melchers
SPF169	V181-51	*atrR*Δ::*ptrA*	This study
AfS35	D141	*akuA*::*loxP*	FGSC
SPF92	AfS35	wt *cyp51A* hph	13
SPF200	AfS35	mSRE *cyp51A* hph	This study
SPF202	AfS35	mATRE *cyp51A* hph	This study
SPF204	AfS35	mHXRE *cyp51A* hph	This study
SPF94	AfS35	TR34 *cyp51A* hph	13
SPF206	AfS35	mdSRE TR34 *cyp51A* hph	This study
SPF208	AfS35	mdATRE TR34 *cyp51A* hph	This study
SPF210	AfS35	mHXRE TR34 *cyp51A* hph	This study
SPF212	AfS35	mpSRE TR34 *cyp51A* hph	This study
SPF214	AfS35	mpATRE TR34 *cyp51A* hph	This study

**TABLE 2:** Plasmids used in this study

Plasmid	Parent	Genotype	Reference
A1	pUC57	wt *cyp51A* hph	5
pSP119	A1	mSRE *cyp51A* hph	This study
pSP120	A1	mATRE *cyp51A* hph	This study
pSP121	A1	mHXRE *cyp51A* hph	This study
L5H	pUC57	TR34 *cyp51A* hph	5
pSP122	A1	mdSRE TR34 *cyp51A* hph	This study
pSP123	A1	mdATRE TR34 *cyp51A* hph	This study
pSP124	A1	mHXRE TR34 *cyp51A* hph	This study
SPF125	A1	mpSRE TR34 *cyp51A* hph	This study
SPF126	A1	mpATRE TR34 *cyp51A* hph	This study

The presence of AtrR was essential for the normal high-level voriconazole resistance seen in both clinical isolates, irrespective of the TR34 or TR46 nature of the *cyp51A* promoter. The overexpression of Cyp51A was also eliminated from these strains when *atrR* was deleted.

Together, these data illustrate the unexpected complexity of the TR34 promoter region in *cyp51A* expression. We argue that a simple increase in dosage of a positively acting region of 34 bp cannot explain the unique behavior of the TR34 promoter. The distal 34-bp repeat behaves positively, but the proximal 34-bp repeat has a strong negative effect on TR34 promoter activity. We hypothesize the presence of a multivalent corepressor (Fig. 2C) that must be engaged by SrbA and AtrR, along with CBC and HapX, to normally repress *cyp51A* transcription. A single transcription factor acting as both a repressor or activator has been extensively documented for mammalian nuclear receptors (15). Loss of the binding sites for SrbA or AtrR strongly activates *cyp51A* expression in the absence of drug induction but only in the context of a duplication of the *cyp51A* promoter. Importantly, neither the TR34 or TR46 duplication includes both the CBC and HapX binding sites, suggesting that these must be lost in order to provide the proper context for the upstream repeat to induce *cyp51A* expression. In the wild-type *cyp51A* promoter, mutations in either the SRE or the ATRE cannot hyperactivate since these elements are also required for normal expression. AtrR is required for voriconazole resistance and Cyp51A overproduction from TR34 and TR46 promoter-driven *cyp51A* genes, and as seen earlier with SrbA (16), AtrR is a crucial determinant for azole resistance in clinical isolates of A. fumigatus.

10.1128/mbio.00702-22.1 TEXT S1 Supplemental materials and methods. Download Text S1, PDF file, 0.04 MB. Copyright © 2022 Paul et al. 2022 Paul et al. https://creativecommons.org/licenses/by/4.0/ This content is distributed under the terms of the Creative Commons Attribution 4.0 International license.
