# Supplementary material for: Differential Functions of Individual Transcription Factor Binding Sites in the Tandem Repeats Found in Clinically Relevant cyp51A Promoters in Aspergillus fumigatus
Source: mBio. 2022 Apr 25;13(3):e00702-22. doi: 10.1128/mbio.00702-22 (PMC9239056; doi:10.1128/mbio.00702-22)
Supplement: FIG S1 [file mbio.00702-22-sf001.pdf]

Supplementary Figures and Material and Methods

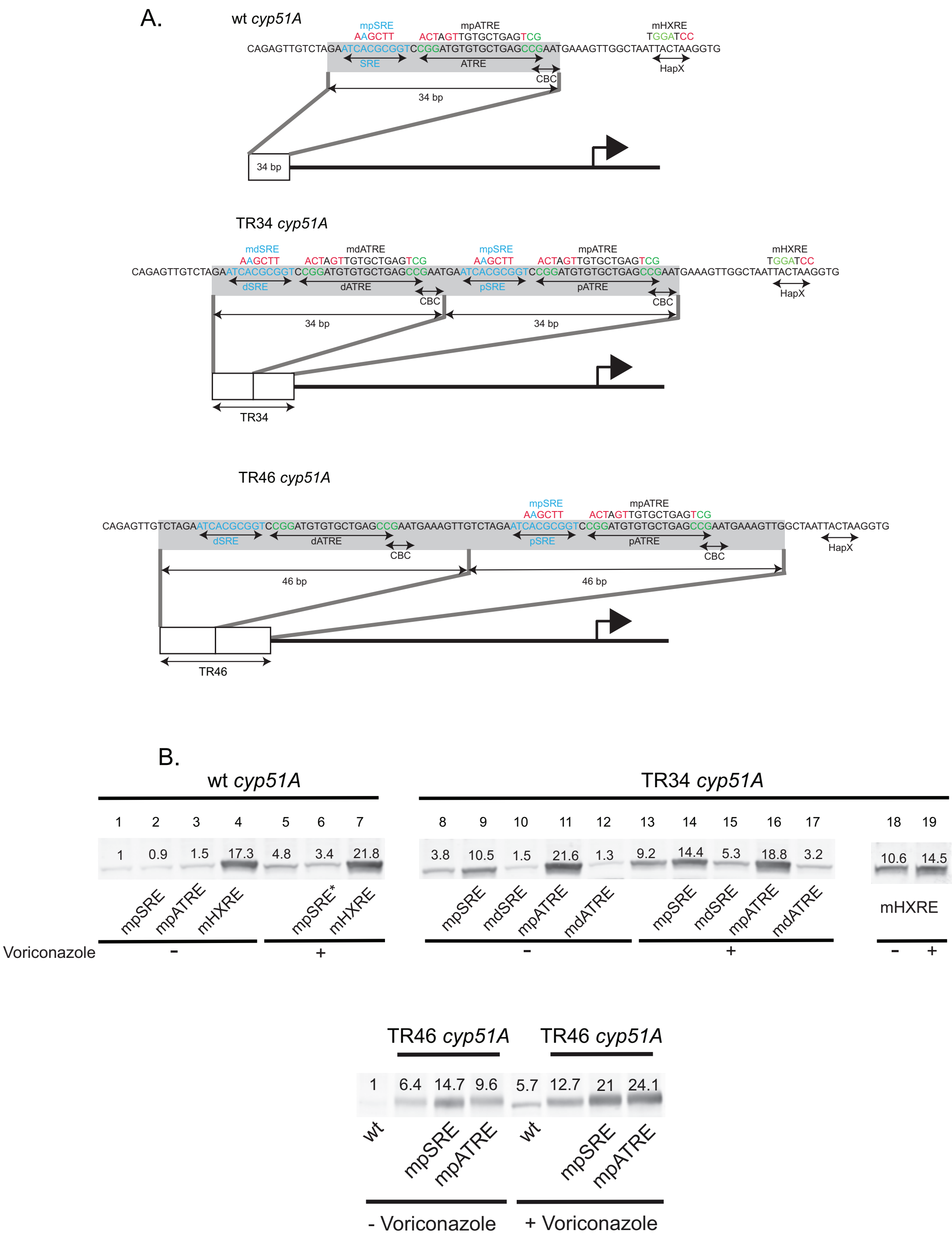

Supplementary Figure 1. **Detailed map of *cyp51A* promoter mutations and analysis of Cyp51A protein levels in response to these alterations**. A. The DNA sequence of the

cyp51A promoter region of interest in this study is shown. The wild-type promoter is shown on the top and the TR34 equivalent is shown on the bottom. Location of the core binding elements for each transcription are indicated below the DNA sequences. Mutant bases are shown in red lettering in each site. Extent of the 34 bp repeat is shown by the gray highlighting. B. Whole cell protein extracts were prepared and analyzed by western blotting using the anti-Cyp51A antiserum (13). Strains lacking the pATRE in the wild-type *cyp51A* promoter context were unable to be grown in the presence of voriconazole and are absent from that analysis. Lanes are numbered at the top of each panel and the numbers near each Cyp51A polypeptide correspond to the quantitation for this experiment. A representative experiment of at least two is shown and expression levels were all normalized back to the wild-type Cyp51A with no drug treatment.
