# Supplementary material for: Differential Functions of Individual Transcription Factor Binding Sites in the Tandem Repeats Found in Clinically Relevant cyp51A Promoters in Aspergillus fumigatus
Source: mBio. 2022 Apr 25;13(3):e00702-22. doi: 10.1128/mbio.00702-22 (PMC9239056; doi:10.1128/mbio.00702-22)
Supplement: TEXT S1 [file mbio.00702-22-s0001.pdf]

Supplementary Materials and Methods

Strains & growth conditions. The lab strains used in this study were derived from the AfS35 (FGSC #A1159). Strains used here are listed in Table 1. *A. fumigatus* strains were typically grown at 37°C in rich medium (Sabouraud dextrose: 0.5% tryptone, 0.5% peptone, 2% dextrose [pH 5.6]). Selection of transformants and the drug disc diffusion assay was performed in minimal medium (MM: 1% glucose, nitrate salts, trace elements, 2% agar [pH 6.5]); trace elements, vitamins, and nitrate salts, supplemented with 1% sorbitol and either 200 mg/liter hygromycin Gold (InvivoGen) or 0.1 mg/litre pyrithiamine. For solid medium, 1.5% agar was added.

Transformation and generation of *A. fumigatus cyp51A* mutants. The plasmid backbone for generating *A. fumigatus cyp51A* promoter mutants was provided by Eveline Snelders and colleagues. This plasmid has a wild-type *cyp51A* promoter, gene and transcription terminator followed by a hygromycin resistance cassette (hph) and 1.3 kb downstream of *cyp51A* for homologous targeted integration (5). The specific *cyp51A* promoter mutations were synthesized as DNA fragments and obtained from GenScript USA Inc. The DNA fragments were then subcloned into the vector backbone by digesting the vector and synthesized DNA fragments with PmlI and BglII. SRE, ATRE and HXRE mutations were marked with HindIII, SpeI and BamH1 restriction sites, respectively. The recombinant plasmids were verified by PCR amplifying a 1kb region around the mutation sites, and restriction digests using the appropriate restriction enzymes. The recombinant plasmids carrying the different *cyp51A* promoters were digested with PmlI and PstI to release the *cyp51A* promoter-gene-hph cassette for transformation. The list of plasmids used for this study is listed in Table 2.

Transformation was performed using in vitro-assembled cas9-guide RNA-ribonucleoproteins coupled with 50 bp microhomology repair templates (19). All these mutant promoters were returned to the native *cyp51A* chromosomal position. Transformants with targeted integration were confirmed by diagnostic PCR of the novel downstream junction as well as by PCR amplification and subsequent sequencing of the *cyp51A* promoter region to confirm the integrity of the promoter region duplication and/or mutation. The single copy nature of *cyp51A* in the targeted integrants was confirmed using qRT-PCR, as described in (13).

Drug Disc Diffusion Assay. Fresh spores of *A. fumigatus* were suspended in 1X phosphate-buffered saline (PBS) supplemented with 0.01% Tween 20 (1X PBST). The spore suspension was counted using a hemocytometer to determine the spore concentration. Spores were then appropriately diluted in 1X PBST. For the drug diffusion assay, ~10^6 spores were mixed with 10 ml soft agar (0.7%) and poured over 15 ml regular agar (1.5%) containing minimal medium. A sterile paper disk was placed on the center of the plate, and 10 μl of 2 mg/liter voriconazole was spotted onto the filter paper for analysis of wild-type and TR34 promoter mutant-containing strains. The same protocol was followed by 10 μl of 3.2 mg/liter voriconazole was spotted onto the filter disk for the analysis of the TR46 promoter-containing strains. The plates were incubated at 37°C and scanned after 72 hours.

Western Blotting. Western blotting was performed as described in (13). The Cyp51A peptide polyclonal antibody used here has been detailed in the reference above, and was used at a 1:500 dilution.

Measurement of mRNA level. Reverse transcription quantitative PCR (RT-qPCR) was performed as described in reference (13), with the following modification. The Ct value of the

gene coding for *tef1* was used for normalization of variable cDNA levels to determine the fold difference in transcript levels with respect to *cyp51A*.
